# *In Vitro* Intracellular Trafficking of Virulence Antigen during Infection by *Yersinia pestis*

**DOI:** 10.1371/journal.pone.0006281

**Published:** 2009-07-17

**Authors:** Tracy L. DiMezzo, Gordon Ruthel, Ernst E. Brueggemann, Harry B. Hines, Wilson J. Ribot, Carol E. Chapman, Bradford S. Powell, Susan L. Welkos

**Affiliations:** 1 Bacteriology Division, United States Army Medical Research Institute of Infectious Diseases (USAMRIID), Fort Detrick, Maryland, United States of America; 2 Integrated Toxicology, United States Army Medical Research Institute of Infectious Diseases (USAMRIID), Fort Detrick, Maryland, United States of America; University of Birmingham, United Kingdom

## Abstract

*Yersinia pestis*, the causative agent of plague, encodes several essential virulence factors on a 70 kb plasmid, including the *Yersinia* outer proteins (Yops) and a multifunctional virulence antigen (V). V is uniquely able to inhibit the host immune response; aid in the expression, secretion, and injection of the cytotoxic Yops via a type III secretion system (T3SS)-dependent mechanism; be secreted extracellularly; and enter the host cell by a T3SS-independent mechanism, where its activity is unknown. To elucidate the intracellular trafficking and target(s) of V, time-course experiments were performed with macrophages (MΦs) infected with *Y. pestis* or *Y. pseudotuberculosis* at intervals from 5 min to 6 h. The trafficking pattern was discerned from results of parallel microscopy, immunoblotting, and flow cytometry experiments. The MΦs were incubated with fluorescent or gold conjugated primary or secondary anti-V (antibodies [Abs]) in conjunction with organelle-associated Abs or dyes. The samples were observed for co-localization by immuno-fluorescence and electron microscopy. For fractionation studies, uninfected and infected MΦs were lysed and subjected to density gradient centrifugation coupled with immunoblotting with Abs to V or to organelles. Samples were also analyzed by flow cytometry after lysis and dual-staining with anti-V and anti-organelle Abs. Our findings indicate a co-localization of V with (1) endosomal proteins between 10–45 min of infection, (2) lysosomal protein(s) between 1–2 h of infection, (3) mitochondrial proteins between 2.5–3 h infection, and (4) Golgi protein(s) between 4–6 h of infection. Further studies are being performed to determine the specific intracellular interactions and role in pathogenesis of intracellularly localized V.

## Introduction

*Yersinia pestis*, the causative agent of bubonic, septicemic, and pneumonic plague, is a globally distributed pathogen [1], [2]. There currently is no licensed human vaccine that elicits effective immunity to pneumonic plague, a severe and highly fatal form of the infection. Greater knowledge of events and interactions supporting infection and the pathogenesis will lead to the development of vaccines and therapeutics that are effective against both bubonic and pneumonic forms of plague.

Research in pursuit of these goals has revealed that fully virulent strains of *Y. pestis* possess several plasmid-encoded proteins that are major immunogens and/or virulence factors, including the *Yersinia* outer proteins (Yops) and virulence antigen (V), which are found on the 70 kb low-calcium response plasmid (pLcr) or pCD1 [3]. These proteins are essential for survival of the organism in mammalian hosts [4]–[12]. The 10 kb pPst (pesticin) or pPCP plasmid encodes plasminogen activator (Pla), a protein that is critical to the establishment of systemic infection from a peripheral site and is also thought to have an essential role in the development of pneumonic plague [13]–[17]. The Yops are induced by growth at 37°C under low-calcium conditions, or by contact with host cells, and are delivered via the multi-component type III secretion system (T3SS) [12], [18]–[21]. These effector proteins function to (1) disrupt cellular processes such as phagocytosis via actin depolymerization (YopE, YopH, YopO, YpkA and YopT), (2) suppress cytokine production and induce apoptosis (YopJ), or (3) resist innate immunity (YopM) [22], [23].

The highly immunogenic pLcr-encoded V is essential to the virulence of *Y. pestis* and required for production of disease, making it a major target for vaccine development [1], [18], [21], [24]–[28]. V promotes infection by suppressing the host's ability to produce inflammatory cytokines, recruit inflammatory cells, and form granulomas in response to infection. Furthermore, V stimulates production of anti-inflammatory cytokines such as interleukin (IL)-10 [21], [25], [26], [29], [30]. As a structural component of the “injection” apparatus, or injectisome, V also is a regulatory protein required for the expression, secretion, and entry of the cytotoxic Yops into host cells by the T3SS [19], [31], [32]. In addition, V is the only pLcr-encoded protein known to be secreted in large amounts into the surrounding medium by yersiniae in contact with eukaryotic cells, and is shown to offer significant protection against plague challenge when given by itself to mice [19], [20], [24]–[26], [29]. Alternatively, V can enter cells by a novel T3SS-independent mechanism [34]. However, its activity, trafficking, and interactions inside host cells are not known. Further research is needed to identify the enzymatic activities, host cell targets, and specific immunosuppressive effects of this complex virulence protein. The objectives of these studies were to discern the mechanism of the T3SS- independent entry of V into the cell and to determine its intracellular course. These goals prompted our effort to identify possible intracellular host proteins targeted by V using immuno-microscopy, density gradient centrifugation, and flow cytometry co-localization studies. Because V appears to localize both intracellularly and systemically in the host, compounds that effectively block the interaction of V and its intracellular protein target(s) could also be effective in impeding or preventing the overall disease progression.

## Results

### Intracellular trafficking of V antigen as detected by microscopy

**Figure 1:**
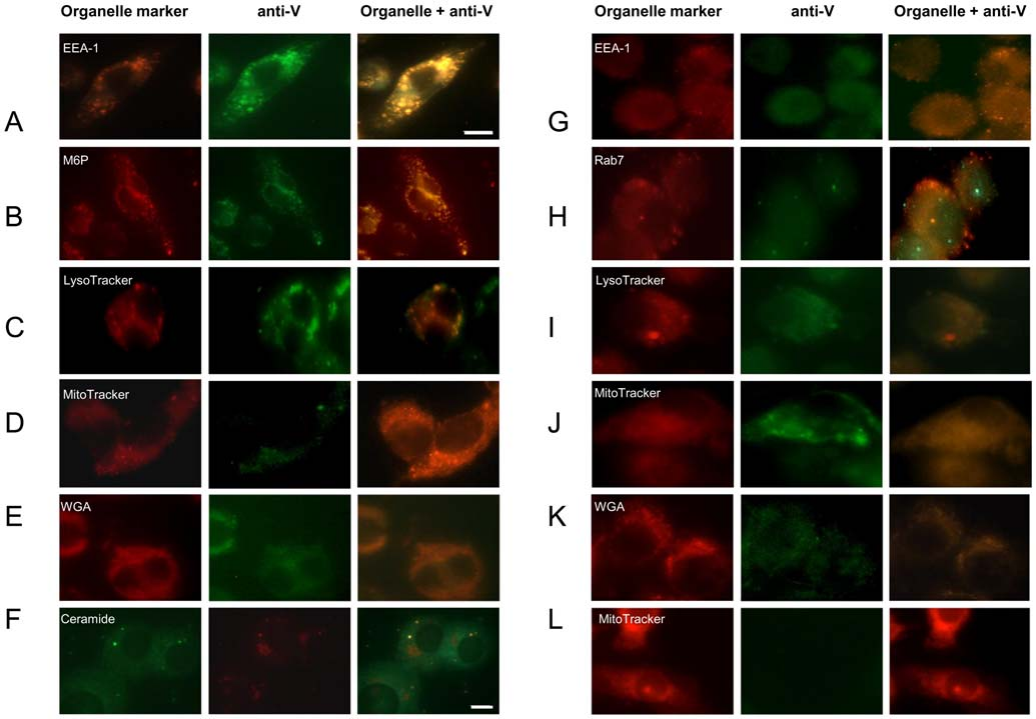
Trafficking of V as detected by IFM. MФs were infected with *Yersinia* for different periods of time. The infecting *Yersinia* strain was C092 pPst- pgm- (panels A–F, H and I), Y pIII p(IB604) (panels G, J, and K); or YpIII p(IB19) (panel L). In panels H and I, SMI of T3S were added to CO92 pPst- pgm- prior to infection. IFM images depicted infected, cytoD-pretreated MΦs stained with organelle markers (Table 1), including EEA-1 Ab (panels A and G) at a 10 min infection, M6P Ab (panel B) or rab7 Ab (panel H) at a 45 min infection, LysoTracker (panels C and I) at a 1 h infection, MitoTracker (panels D, J, and L) at 3 h infections, and/or anti-V Ab at all time points. MФs were infected for 4 h, stained with BODIPY ceramide (panel F) or WGA (panel E and K) after 4 h of infection, followed by anti-V staining. Co-localization was defined by yellow/orange regions, suggesting the presence of green and red fluorescence, which was observed at different sites during the course of infection, i.e., in early endosomes after 10 min, late endosomes after 30–45 min, lysosomes after 1–2 h, mitochondria after 2.5–3 h, and the Golgi apparatus after 4 h. Scale bar = 10 micrometers (µm) (panels A–E, and G–L) and 5 µm (F).

The entry of V into eukaryotic cells, which was originally demonstrated using HeLa cells infected with *Y. pestis* KIM strains [34], was corroborated using cells infected with the *Y. pseudotuberculosis* V-producing recombinant Y. ptb. pTrcV strain. HeLa cells infected with Y. ptb. pTrcV were stained with a fluorescent fluorescein (FITC)-labeled anti-V Ab, whereas uninfected cells and cells infected with the parent V-negative YpIII p(IB19) strain showed only background levels of fluorescence, confirming the internalization and specific staining of V (data not shown). Several different approaches were then used to monitor the intracellular movement of V during the course of infection. Time-course experiments with samples collected at intervals over 6 h were performed with murine J774A.1 (J774) MΦs pretreated with cytochalasin D (cytoD) to prevent phagocytosis [34], [35]. Then MΦs were infected with V antigen-producing *Yersinia* strains, including Y. ptb. pTcrV and *Y. pestis* strain CO92 pPst- pgm-, each of which have an intact T3SS. Thus, some of the V detected intracellularly may have been the result of *Yersinia* secretion complex (Ysc)-associated V, based on findings of Fields and Straley [34]. Therefore, to more specifically characterize the T3SS-independent entry of V, *Y. pseudotuberculosis* strain YpIII p(IB604), which contains a deletion in the *yopB* gene coding for a required component of the T3S apparatus [36], and CO92 pPst- pgm- in the presence of small molecule inhibitors (SMI) of T3S, were used for infection. The presence of Yops was detected by staining with the labeled Abs specific for YopM, YopE, YopH, and YopD (data not shown). Uninfected and infected MΦs were then incubated with fluorescently-labeled Abs to organelle markers (Table 1) or V. V was first detected intracellularly 10 min after infection with CO92 pPst- pgm- (Fig. 1, panel A) or YpIII p(IB604) (Fig. 1, panel G) in early endosomes, as indicated by the co-localization of FITC-conjugated anti-V Ab and early endosomal antigen -1 (EEA-1) Abs (Table 1) in immunofluorescence microscopy experiments (IFM). The antigen could be detected in early endosomes during the first 30 min of infection. Subsequently, the majority of anti-V Ab staining co-localized with that of Abs to late endosomal markers, as represented in Fig. 1 panels B (CO92 pPst- pgm), and H (CO92 pPst- pgm- with SMI), using M6P and rab7 respectively, after 30–45 min of infection. V appeared in lysosomes between 1 and 2 h after infection, as identified by the overlap in staining of LysoTracker and anti-V Ab, as illustrated for CO92 pPst- pgm- (Fig. 1, panel C), and SMI-pretreated CO92 pPst- pgm- (Fig. 1, panel I) infected MΦs. Afterward, V was observed in mitochondria, as identified by the overlap in MitoTracker and anti-V Ab staining, from 2.5–3 h of infection, as seen in Fig. 1. panels D (CO92 pPst- pgm-) and J (YpIII p[IB604]). Similar results were observed with SMI-pretreated CO92 pPst- pgm- and Y. ptb. pTcrV infected MΦs under the same conditions (data not shown). Furthermore, Ab co-localization experiments showed that after 4 h, V had associated with Golgi apparatus markers in MΦs, including β-cop (data not shown), BODIPY ceramide (Fig. 1, panel F [CO92 pPst- pgm-]), golgin-97 (data not shown), or with trans-Golgi marker WGA in J774 MΦs (Fig. 1, panels E [CO92 pPst- pgm-] and K [YpIII p{IB604}]). V remained in the Golgi, as monitored by the co-localization of the Golgi markers (Table 1) and anti-V Ab, until after 6 h of infection, when decreasing cell viability prevented the determination of localization. Conversely, MΦs infected with the V-negative strain YpIII p(IB19) showed no overlap of staining with anti-V Ab and the organelle markers, including MitoTracker, after 3 h of infection (Fig. 1, panel L). In addition, no observable overlap in staining between anti-V Ab with catalase, histone H1, CellTracker Orange, ER-Tracker Red dye, or calreticulin Ab (data not shown) (Table 1) was detected at any of the time intervals tested. These results suggested V did not co-localize to the peroxisomes, nuclei, cytoplasm, or endoplasmic reticulum (ER), respectively.

**Table 1:** Organelle-associated antibodies and markers.

Organelle	Marker	Abbreviation	Source	Secondary
Cytoplasm	CellTracker Orange CMRA	CellTracker	Invitrogen	n/a
Endoplasmic Reticulum	anti-calreticulin	n/a	Abcam	rhodamine
	ER-Tracker BODIPY^b^ TR	ER-Tracker Red	Invitrogen	n/a
	ER-Tracker Green FL	ER-Tracker Green	Invitrogen	n/a
Endosome	anti-early endosome antigen-1	EEA-1	Abcam	rhodamine
	anti-mannose-6-phosphate receptor	M6P	Abcam	FITC
	anti-rab7	Rab 7	Abcam	rhodamine
Golgi	βeta-cotamer protein	β-cop	Abcam	rhodamine
	Green-fluorescent BODIPY^b^ FL C5 ceramide	Brefeldin A	Invitrogen	n/a
	Brefeldin A BODIPY b 568/668	Ceramide	Invitrogen	n/a
	anti-Golgin-97	Golgin-97	Invitrogen	n/a
	Alexa Fluor 594 wheat germ agglutinin	WGA	Invitrogen	n/a
Lysosome	anti-lysosome-associated membrane protein-1	Lamp-1	Abcam	rhodamine
	anti-lysosome-associated membrane protein-2	Lamp-2	Abcam	rhodamine
	LysoTracker Red DND-99	LysoTracker	Invitrogen	n/a
Mitochondria	MitoTracker Red CXRos	MitoTracker	Invitrogen	n/a
	anti-voltage-dependent anion channel-1	VDAC-1	Abcam	rhodamine
Nucleus	4′, 6-diamidino-2-phenylindole	DAPI	Sigma	n/a
	H1 histone	n/a	Novus	rhodamine
Peroxisome	anti-catalase	Catalase	Abcam	rhodamine

a Not Applicable.

b Boron Dipyromethene Difluoride.

**Figure 2:**
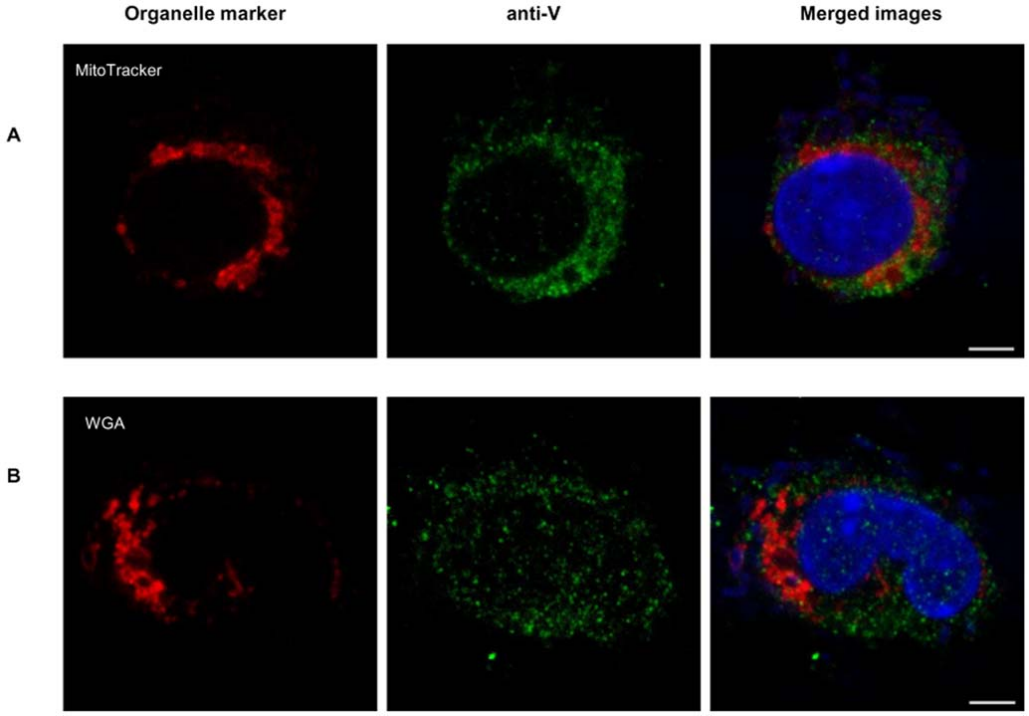
Trafficking of V as detected by ICM. (A) MΦs were infected for 3 h with CO92 pPst- pgm- and then stained with MitoTracker and FITC conjugated anti-V Ab followed by Hoechst 33342 dye. (B) MΦs were stained with Alexa Fluor 594-labeled WGA, infected with CO92 pPst- pgm- for 4 h, and stained with FITC anti-V Ab, followed by secondary staining with FITC-labeled anti-mouse IgG, and tertiary staining with Alexa Fluor 488 labeled anti-rabbit IgG Abs. MΦs were also stained with Hoechst 33342 Dye. All images were captured with a BioRad 2000MP multiphoton confocal system attached to a Nikon TE300 inverted microscope and merged in last panel. Co-localization was defined by an overlap of red and green staining, suggesting V was present in mitochondria and Golgi, after 3 and 4 h of infection, respectively. Scale bar = 5 µm.

In addition to IFM, immunoconfocal microscopy (ICM) performed on MΦs infected for 3 h with CO92 pPst- pgm- demonstrated the staining of individual mitochondria with MitoTracker (Fig. 2, panel A, column 1), and of V antigen with FITC-conjugated anti-V (Fig. 2 panel A, column 2). Merging of the images suggested the presence of V in individual mitochondria (Fig. 2, panel A, column 3), a finding which was in agreement with IFM experiments. When MΦs were infected for 4 h and stained with anti-V Ab or WGA, the resulting merged ICM images demonstrated a perinuclear association of V in the trans-Golgi network (Fig. 2, panel B), supporting our previous IFM results. As a control for staining, uninfected MΦs were incubated with FITC anti-V, FITC-labeled anti-mouse IgG, and Alexa Fluor 488 labeled anti-rabbit IgG Abs. Infected MΦs were incubated with FITC anti-mouse/Alexa Fluor 488 anti-rabbit Abs. Only background levels of staining were observed (data not shown).

To determine if V was trafficking from lysosomes to mitochondria and Golgi sequentially or simultaneously, MΦs were pretreated with cytoD, Ru360 (an inhibitor of mitochondrial function), or both for 30 min before infection with YpIII p(IB604). MΦ pretreatment with Ru360 in the presence of cyto D did not appear to alter V's localization to the mitochondria after 2.5–3 h of infection, as monitored by anti-V Ab and MitoTracker staining (data not shown). After 4 h of infection in the presence of cyto D alone, MΦs stained with WGA and then by FITC anti-V Ab demonstrated co-localization of V with Golgi protein(s) (Fig. S1, panel A), as observed in Fig. 1 (panels E and K. However, in the presence of Ru360 alone, V staining was intensely distributed throughout the cells, making it difficult to observe discrete MΦs (Fig. S1, panel B). When both inhibitors were present, no anti-V staining was observed (Fig. S1, panel C), results which may suggest that V traffics from the mitochondria to the Golgi in succession.

Co-localization of anti-V Ab with organelle-markers was analyzed quantitatively in JACoP, an Image J plugin, using Pearson's correlation coefficients (PCCs) and Mander's overlap coefficients (MOCs), after correcting for background. For endosomal marker EEA-l Ab, PCCs were calculated to be r = 0.959 (CO92 pPst- pgm-) and r = 0.928 (YpIII p[IB604]), after 10 min of infection. These results suggested that co-localization occurred between anti-V and EEA-1 Abs (Table 2), since co-localization increases as the coefficients approach 1 [37], [38]. The r^2^ values expressed as percentages indicated that 92.0% of the MΦs infected with CO92 pPst- pgm- and 86.1% of MΦs infected with YpIII p(IB604) demonstrated co-localization between EEA-1 and anti-V Ab staining. Corresponding results derived using the MOC on the same images indicated r = 0.941 (CO92 pPst- pgm-) and r = 0.946 (YpIII p[IB604]). This analysis was repeated for M6P, rab7, LysoTracker, MitoTracker, and WGA at 45 min, 1 h, 3 h, and 4 h respectively (Table 2), yielding results that indicated the majority of anti-V Ab staining overlapped with the staining of each of these organelle-associated markers. PCCs of r = 0.017 and r = 0.126 were calculated for CO92 pPst- pgm- or YpIII p(IB604) infected MΦs, respectively, (Table 2), expressing less than 1% co-localization.

**Table 2:** Co-localization analysis of IFM intracellular V trafficking.

Organelle	Strain	Pearson's (r = )	Mander's Overlap (r = )
**EEA-1**	*CO92 pPst- pgm-*	0.959	0.941
	*YpIII p(IB604)*	0.928	0.946
**M6P**	*CO92 pPst- pgm-*	0.983	0.979
	*YpIII p(IB604)*	0.974	0.970
**Rab7**	*CO92 pPst- pgm-*	0.921	0.941
	*YpIII p(IB604)*	0.908	0.926
**LysoTracker**	*CO92 pPst- pgm-*	0.871	0.880
	*YpIII p(IB604)*	0.846	0.841
**MitoTracker**	*CO92 pPst- pgm-*	0.951	0.957
	*YpIII p(IB604)*	0.964	0.925
**WGA**	*CO92 pPst- pgm-*	0.852	0.864
	*YpIII p(IB604)*	0.888	0.886
**Catalase**	*CO92 pPst- pgm-*	0.017	0.001
	*YpIII p(IB604)*	−0.126	0.005

To determine at an ultrastructural level if V was present in the early endosomes, MΦs were infected with CO92 pPst- pgm- for 15 min, 30 min, and 3 h, followed by staining with gold labeled anti-V Ab (6 nanometers [nm]) and 15 nm gold-labeled Abs to EEA-1, M6P, or VDAC-1 for immunoelectron microscopy (IEM) analysis. Anti-V Ab gold particles were found in close proximity to EEA-1 and M6P Abs near the MΦ cell membrane, supporting the IFM observations (data not shown). Likewise, after 3 h of infection, IEM data also showed that V antigen localized to mitochondria, marked by VDAC-1 (data not shown).

### Intracellular trafficking of V antigen as detected by gradient fractionation and immunoblotting

**Figure 3:**
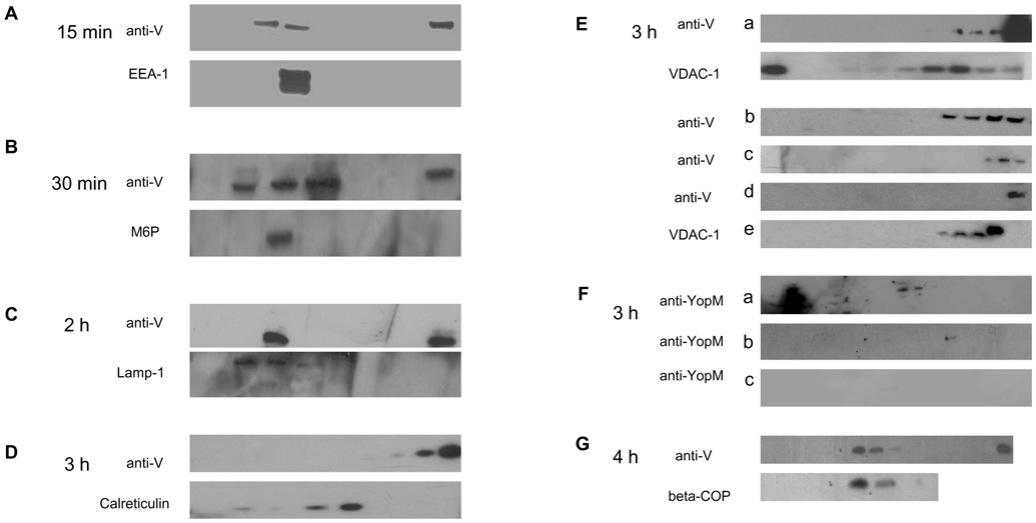
Trafficking of V as detected by density gradient centrifugation and immunoblot analysis. MФs were infected with CO92 pPst- pgm- (panels D-E-a, F-a-b, and G), SMI-pretreated CO92 pPst- pgm- (panels B, C, and F-c), or YpIII p(IB604) (panels A, E-c and E-e) for 15 min, 30 min, 2 h, 3 h, or 4 h. The fractions, or purified V (approx. 35 kD), were immunoblotted with anti-V Ab or with organelle-associated markers. The latter included Abs to EEA-1 (180 kD), M6P (31 kD), Lamp-1 (120 kD), VDAC-1 (31 kD), calreticulin (∼63 kD) and β-COP (81 kD). After infection for 15 min (panel A), a band recognized by the anti-V mAb was observed in fractions that also contained bands detected by EEA-1 Ab. Similar patterns were observed for anti-V and Abs to: (panel B) M6P after 30 min of infection, (panel C) Lamp-1 after 2 h, (panels E) VDAC-1 after 3 h, and (panel G) β-COP after 4 h. At all time intervals tested, calreticulin (∼63 kD) was not observed immunologically in fractions recognized by anti-V; a representative blot is shown in panel (D). MΦs were also infected with for 3 h with Y. ptb. pTcrV (Panel E-b) or with the V-negative strain YpIII p(IB19) for 3 h (Panel E-d). In panel F, immunoblots were developed using anti-YopM Ab, to detect YopM in MФs that were not treated with cytoD (Panel F-a), treated with cytoD (Panel F-b), or treated and infected with SMI treated CO92 pPst- pgm- (Panel F-c) are shown.

Lysates of uninfected MΦs and MΦs infected for various times were fractionated by centrifugation and evaluated by gel immunoelectrophoresis. After 15 min of infection with YpIII p(IB604) (Fig. 3, panel A), a band recognized by anti-V mAb was observed in the fraction that also was detected by EEA-1 Ab (180 kD). Fractions that revealed bands when stained with anti-V Abs also demonstrated staining with (1) late endosomal antigen M6P (31kD) after 30 min of infection with SMI-pretreated CO92 pPst- pgm- (Fig. 3, panel B), (2) lysosomal marker Lamp-1 (120 kD) after 2 h with SMI-pretreated CO92 pPst- pgm- (Fig. 3, panel C), (3) mitochondria protein VDAC-1 (31 kD) after 3 h (Fig. 3, panel E) with (a) CO92 pPst- pgm-, (b) Y. ptb. pTcrV, or (c) YpIII p(IB604), and (4) Golgi marker β-COP after 4 h infection with CO92 pPst- pgm- (Fig. 3, panel G). However, fractions immunoblotted with the organelle-associated markers described in Table 1 did not demonstrate bands in fractions stained with anti-V Abs at any other time intervals tested. Representative blots (Fig. 3, panel D) are depicted, showing anti-V Ab staining in the latter fractions, i.e. heavier fractions, which did not align with the calreticulin Ab (∼63 kD) stained bands, after 3 h of infection. As shown in Fig. 3, panel F, MФs that were not pretreated with cytoD and were infected with CO92 pPst- pgm-, revealed bands corresponding to anti-YopM Ab staining (panel F-a), the quantity of which was reduced by the addition of cytoD (panel F-b), and eliminated when CO92 pPst- pgm- was pretreated with SMI before infection (panel F-c). Additionally, none of the fractions demonstrated staining with anti-Yop D, anti-Yop E, or anti-Yop H Abs when SMI of T3S were present (data not shown). In contrast, MΦs infected for 3 h with YpIII p(IB19) yielded no fractions recognized by anti-V Ab (Fig. 3, panel E-d) on the immunoblots, even though mitochondrial proteins were present (Fig. 3, panel E-e). Thus, the results suggested that early and late endosomal proteins were present in fractions containing V after 15–30 min of infection. Subsequently, V was present in fractions containing lysosomes after 1 h (data not shown) and 2 h, mitochondria after 3 h, and cis-Golgi after 4 h, as determined by anti-V Ab and organelle-associated marker immunoblotting. However, V does not appear to have localized to fractions containing ER, nuclei, or cytoplasm (as suggested by IFM), or peroxisomes at any of the time intervals tested.

### Intracellular trafficking of V antigen as detected by flow cytometry

**Figure 4:**
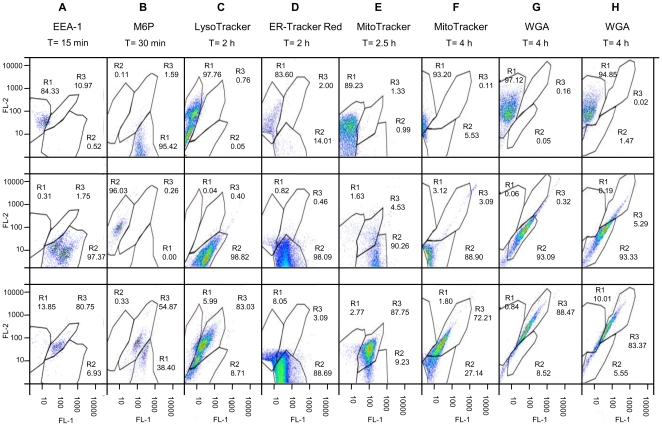
Trafficking of V as detected by flow cytometry. MФs were infected with either CO92 pPst- pgm- (panels A–E, G) or Y.pIII pIB604 (panel H); alternatively, LFnV (Panel F) was delivered with PA for different periods of time. Panel (A): MФs were infected for 15 min and stained with EEA-1 and anti-V Abs. Panel (B): MФs were infected for 30 min, stained with M6P and anti-V Abs. Panels (C and D): MФs were infected for 2 h followed by staining with LysoTracker or ER-Tracker Red, respectively, and anti-V Ab. Panel (E): MФs were infected for 2.5 h, stained cells with MitoTracker, followed by anti-V Ab. Panel (F): MФs were exposed to LFnV for 4 h, stained with MitoTracker, followed by anti-V Ab. Panel (G and H): MФs were stained for 30 min with WGA, infected for 4 h, and stained with anti-V Ab. Co-localization was defined by a population shift to region 3.

Uninfected and infected samples were lysed, stained with anti-V and/or organelle-associated markers, and analyzed by flow cytometry. Fluorescence was plotted as FL-1 (green) vs. FL-2 (red) channel pseudocolor dot plots in FlowJo (version 7.1.2). Two populations, one each representing FL-1 and FL-2 channel detected fluorescence, were observed when no co-localization was present. A single population detected by both the FL-1 and FL-2 channels was considered to represent co-localization. For all experiments anti-V/FITC Abs (FL-1 channel) were used, expect for M6P Ab stained samples, which were stained with unconjugated anti-V mAb followed by rhodamine conjugated goat anti-mouse IgG and were detected in the FL-2 channel. Organelles isolated from MΦs infected with CO92 pPst- pgm- for 15 min were stained with FITC-anti-V Ab and Abs to organelle markers. Analysis on a BD FACSCalibur Flow Cytometer revealed a single population detected by the FL-2 channel (84.33%) when the organelles were stained with EEA-1 Ab alone (Fig. 4, panel A row 1). When stained with anti-V Ab alone, 97.87% of the organelle population represented FL-1 fluorescence (Fig. 4, panel A, row 2). However, when EEA-1 and anti-V Abs were both present, a single population (80.75%) was detected by both channels (Fig. 4, panel A, row 3), suggesting co-localization. Thus, the results confirmed earlier IFM and immunoblot experiments that showed an association of V with early endosomes during the first 10–15 min of infection. Similar finding were observed when organelles were infected with CO92 pPst- pgm- or YpIII p(IB604) and stained with the associated organelle-markers at the time intervals established in the IFM and immunoblotting experiments. Representative plots of CO92 pPst- pgm- (Fig. 4, panels A-E, and G) and YpIII p(IB604) (Fig. 4, panel H) are shown, stained with anti-V Abs and (1) M6P Ab/FITC goat anti-chicken after 30 min (Fig. 4, panel B), (2) LysoTracker after 2 h (Fig. 4, panel C), (3) MitoTracker after 2.5 h (Fig. 4, panel G), and (4) WGA after 4 h of infection (Fig. 4, panel H). For samples stained with both M6P and anti-V Abs, region 3 (Fig. 4, panel B, row 3) represented the largest percentage (54.87%). This population was detected in both the FL-1 and FL-2 channels, suggesting co-localization of V in the late endosomes. Region 2, which represented the population stained by anti-V alone as recognized by only the FL-2 channel, comprised less than 1% of the total population. These results indicated that almost all of the anti-V Ab staining was accounted for in region 3. However, 38.40% of the late endosomes had no V present, suggested by the population observed in region 1, which was detected in the FL-1 channel alone and corresponded to M6P Ab staining. In contrast to the above findings, no co-localization was observed with anti-V Abs and any of the organelle-associated markers at any other time interval tested, including the endoplasmic reticulum, stained with ER-Tracker Red (Fig. 4, panel D) and peroxisomes, stained with catalase Ab (data not shown). Those MΦs infected with YpIII p(IB19), the V-negative strain, lacked a FL-1 channel population when stained with anti-V Abs.

**Figure 5:**
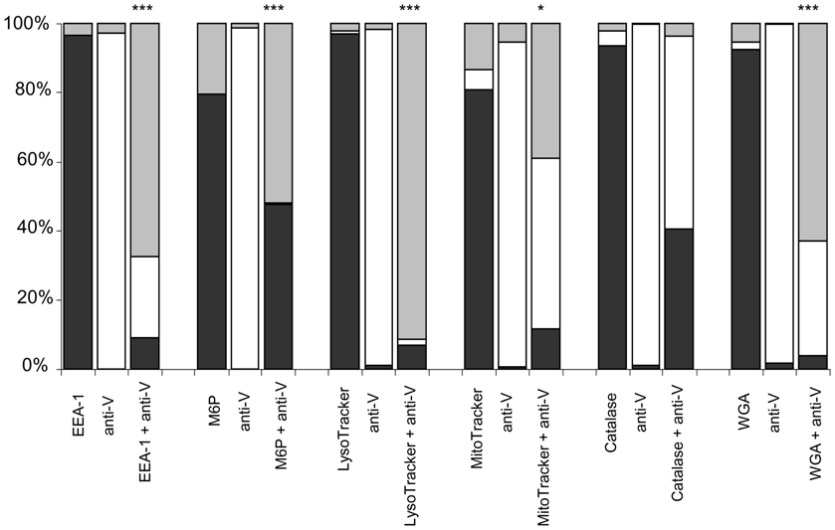
Region statistics of flow cytometry data. Percentages of flow cytometry fluorescence detected in the FL-1 only (region 1), FL-2 only (region 2), or both (region 3) channels, which included infected organelles stained with organelle-associated markers alone, anti-V alone, or both, were plotted. Post ANOVA t-tested indicated significant increases in the percentage of those isolated organelles that demonstrated both organelle and anti-V Ab staining in region 3 as compared to anti-V Ab staining alone, as observed in those organelles stained with both anti-V Ab and EEA-1 Ab (***, P<0.001), LysoTracker (P<0.001), MitoTracker (*, P<0.05), or WGA (P<0.001), suggesting co-localization. No significant increase in a double-stained population was observed when isolated organelles were stained with both catalase and anti-V Abs.

A quantitative analysis based on the percentages of one- and two-stained populations supported our findings, as profiled in Fig. 5. For the three regions plotted, a post ANOVA (analysis of various) t-test revealed statistically significant differences in region 3 between samples stained with anti-V Ab alone, as compared to those stained with both the organelle-associated marker and anti-V Ab for EEA-1 (P<0.001), M6P (P<0.001), LysoTracker (P<0.001), MitoTracker (P<0.05), and WGA (P<0.001) at the specified time intervals. No significant differences (P>0.05) were detected in region 3 when samples were stained with ER-Tracker or catalase and anti-V Abs, as compared to anti-V Ab staining alone. These data substantiate our IFM and immunoblotting results by a third independent method.

As an alternate to infection for the demonstration of V trafficking, the lethal factor (LF) and protective antigen (PA) from *Bacillus anthracis* was used to shuttle V (LFnV-PA system) to MФs, and the interaction was evaluated by flow cytometry. The co-localization of MitoTracker and anti-V Ab was observed after 4 h incubation with LFnV-PA (Fig. 4 panel F).

After 6 h of infection, cell viability decreased significantly, as detected by IFM, and V was not tracked beyond the Golgi apparatus. Thus, flow cytometry was utilized to evaluate any effect of V on cell viability. MΦs were infected with all strains for 4–12 h at intervals of 1 h, including CO92 pPst- pgm- and Y. ptb. pTcrV in the presence/absence of SMI of T3S. Cytotoxicity was measured by propidium iodide (PI)/Syto-13 or DEADRed/Syto-10 staining. A 2-way ANOVA comparison showed that there were significant differences (P<0.05) between live and dead populations of uninfected and infected MФs. Results of statistical analyses indicated that *Yersinia* strains with an intact T3SS were significantly more cytotoxic (P<0.001) after 5 h of infection, as compared to MΦs infected with the V-negative strain, YpIII p(IB19) (Fig. S2). There were no significant differences observed between YpIII p(IB19) infected MΦs and (1) YpIII p(IB604) or (2) CO92 pPst- pgm- in the presence of SMI of T3S, which may indicate V has a minimal role in cellular cytotoxicity. However, there was a significant difference in the cytotoxicity associated with MΦs infected with SMI-pretreated Y. ptb. pTcrV infected compared to that with the YpIII p(IB19) strain.

## Discussion

Using an *in vitro* HeLa infection model, Fields and Straley (1999) showed that V is not only required for the T3SS-mediated host cell targeting and entry of the effector Yops, but that it also is capable of entering these cells by a novel T3SS-independent pathway. During their production and T3SS-facilitated transfer into host cells, the effector Yops (YopE, YopH, YopJ, YopM, YpkA, and YopT) are not secreted into the medium [2], [20]. In contrast, V not only partitions to the target cell cytosol, but also localizes to the bacterial surface where it is an integral component of the T3SS injectisome: Specifically V is found at the tip [32] and in substantial amounts in the extracellular medium. Although it is also contact-activated, the localization of most of the V to the cytosol does not involve the T3SS. This finding as reported by Fields and Straley [34] was evidenced by infections with strains lacking a functional T3SS apparatus or the pLcr plasmid, which encodes the T3SS components. In addition, the V that localizes within the target cell was not the result of secretion into the culture medium. Therefore, the authors concluded that a novel, contact-activated, T3SS-independent pathway exists. This mechanism for host cell transfer of V was hypothesized to involve non-pLcr-encoded proteins that compose a “LcrV-transporting contact-activated translocator (VCAT [34]),” which spans the bacterial surface membranes and then, directly or indirectly, transfers V to the target cell. Little is known about the composition of this translocator, other proteins that might traverse it, the mechanism of eukaryotic host cell internalization, or the function of the internalized V. Our objectives were to identify how V enters the host by T3SS-independent means, determine its trafficking pattern, and understand localization within the cells, so as to ultimately help identify the host protein(s) with which it interacts.

Several assays were developed herein to monitor V over the course of infection of *in vitro* cell cultures. Recently, *Y. pestis* was confirmed to target immune cells, the majority of which are MΦs and neutrophils [39]. As a result, our experiments focused on MΦs infected with the attenuated *Yersinia* strains in the presence of cytoD to prevent bacterial invasion [33], [39], [40]. This *in vitro* model of infection by virulent *Y. pestis* was used to investigate the extent of intracellular V trafficking. *Yersinia* species utilize the Ysc injectisome during T3S, which includes three major components at the tip (YopB, YopD, and LcrV). These proteins allow the cytotoxic Yops to penetrate the host cell [41], [42]. In addition to *Y. pestis* CO92 pPst- pgm-, MΦs were infected with the *Y. pseudotuberculosis* YpIII p(IB604) strain, a mutant with a deletion in the *yopB* gene, which, along with YopD and LcrV, is a critical determinant of the host cell translocation pore. The absence of YopB thus blocks delivery of Yops [36], [43]. This mutant was used to confirm that the V, which we observed intracellularly by microscopy, immunoblotting, and flow cytometry, was translocated by a T3SS-independent mechanism. Using small molecule inhibitors of the T3S we were able to confirm these findings with both *Y. pseudotuberculosis* and *Y. pestis* strains. The presence of V was confirmed in all strains tested (Fig. 3, panels E-a, E-b, and E-c), except the V-negative YpIII p(IB19) (Fig. 3, panel E-d), which showed a single band detected by anti-V Ab only in the lane containing purified V, a positive control. Whereas Yops could also be detected in MΦs infected with the T3SS-proficient CO92 pPst- pgm-, they did not appear to be translocated by the YpIII p(IB604) strain (data not shown) or by the SMI-pretreated CO92 pPst- pgm- strain (Fig. 3, panel F-c). Results of experiments with YpIII p(IB604) and SMI-pretreated CO92 pPst- pgm- confirmed those obtained in the initial experiments with the untreated CO92 pPst- pgm- strain, suggesting that V becomes endocytosed by MΦs and traffics to the mitochondria and Golgi apparatus, in a manner that is independent of Yops translocation.

Our results suggest that V followed an endocytic-associated pathway and co-localized with endosomal proteins between 10–45 min of infection, followed by lysosomal protein(s) between 1–2 h. After 2.5–3 h of infection, V was observed in conjunction with mitochondrial proteins, and subsequently with Golgi protein(s) between 4–6 h of infection. Although we have not yet determined which type of endocytic pathway is involved, the V trafficking sequence we determined, summarized in Table 3, was discerned from the concordance of results from parallel microscopy, immunoblot, and flow cytometry analyses of MΦs infected with V -producing *Y. pestis* and *Y. pseudotuberculosis* strains. Intracellular V trafficking was not observed after 6 h of infection, due to a decrease in cell viability. V may play a role in MΦ viability, since flow cytometry analysis of SMI-pretreated Y. ptb. pTcrV infected MΦs demonstrated significant differences in cell death as compared to MΦs infected with a V-negative strain; however, further research is necessary to clearly define intracellular V's function. Recent preliminary studies were performed using Ru360, which selectively affects mitochondrial Ca^2+^ transport [44], [45], and appeared to prevent the trafficking of V to the Golgi apparatus. This observation suggests that the trafficking that we discerned is sequential and is not a result of simultaneous localization to the mitochondria and Golgi. The intense anti-V Ab staining observed in MΦs pretreated with Ru360 alone was likely the result of V detection on the internalized bacterium's surface (either directly associated or present in association with the T3S system components). Further research will be performed utilizing other inhibitors to confirm our results, although most mitochondrial inhibitors nonspecifically influence other cellular processes or induce apoptosis. In our studies, markers specific to each organelle were selected when possible, but due to the dynamic trafficking and recycling of proteins, not all markers belonged solely to one organelle. Consequently, we chose several markers for each organelle to strengthen the likelihood that the overlap in staining we observed was due to V localization to that organelle. For instance, differentiating late endosomes from lysosomes can be problematic. The M6P receptor antibody was chosen as a marker for late endosomes despite the presence of these receptors in pre-lysosomes [46], [47] and the trans-Golgi network [48]–[50]. Thus, we chose an additional marker, rab7, which is associated with late endosomes [51]–[53]. Despite any potential issues with marker specificity, neither our lysosomal markers (LysoTracker, Lamp-1, and Lamp-2), nor our trans-Golgi marker, WGA, indicated co-localization with anti-V Ab after 30–45 min of infection as was observed with our M6P and rab7 Abs. As a result, we considered V to be present in the late endosomes after 30–45 min of infection.

**Table 3:** Summary of intracellular V trafficking.

Marker	15 min	30 min	45 min	1 h	2 h	3 h	4 h	5 h	6 h
CellTracker									
Calreticulin	−	−	−	−	−	−	−	−	−
ER Tracker	−	−	−	−	−	−	−	−	−
EEA-1	+	+	−	−	−	−	−	−	−
M6P	−	+	+	−	−	−	−	−	−
β-cop	−	−	−	−	−	−	+	+	+
Brefeldin A	−	−	−	−	−	−	+	+	+
Golgin-97	−	−	−	−	−	−	+	+	−
WGA	−	−	−	−	−	−	+	+	+
Lamp-1	−	−	−	+	+	−	−	−	−
Lamp-2	−	−	−	+	+	−	−	−	−
LysoTracker	−	−	−	+	+	−	−	−	−
MitoTracker	−	−	−	−	−	+	−	−	−
VDAC-1	−	−	−	−	−	+	−	−	−
DAPI	−	−	−	−	−	−	−	−	−
H1 histone	−	−	−	−	−	−	−	−	−
Catalase	−	−	−	−	−	−	−	−	−

MΦs infected with the V-negative YpIII p(IB19) strain demonstrated no staining by anti-V Ab in IFM, immunoblots, or flow cytometry analysis, regardless of the organelle-associated marker at any given time point. Finally, the results of the MΦ infection experiments were generally supported by flow cytometry analysis in which V was delivered to the MΦs by the LFnV-PA system. Anthrax PA binds LF, triggering endocytosis of the PA-LF complex which traffics to the endosomes via the endocytic pathway. The toxic protein is then translocated across the endosomal membrane and released into the cytoplasm [54], [55]. The system involving the PA-mediated delivery of a protein fused to the nontoxic LFn moiety into mammalian cells has been exploited to deliver proteins, especially toxins such as diphtheria toxin [56]. V was observed to traffic to the mitochondria after a 4 h incubation with LFnV-PA, as shown by flow cytometry (Fig. 4, panel F). These results differed from those obtained with the usual MФ infection protocol, in which anti-V Ab co-localized with the mitochondrial markers after 2.5–3 h of infection. This variation in the timing of V-host compartment interactions most likely stems from a difference in the kinetics of V trafficking that occurs when V is translocated during a natural infection compared to that by PA-mediated uptake. The kinetics of LFn- mediated internalization and trafficking have not yet been determined. Regardless of these differences, the results of both the infection model and of the PA-mediated transfer system verify that V can enter host cells by a T3SS-independent method and exhibit a specific linear sequence of trafficking within these cells.

Eukaryotic cell processing of internalized material classically involves transport from early to late endosomes, and then to lysosomes where degradation typically occurs. In contrast, internalized bacteria, viruses, and toxins often bind, enter cells, and traffic intracellularly by alternative and distinct pathways [57]–[63]. As discussed by Johannes and Decaudin [59], toxins utilize various pathways of entry (clathrin-dependent and -independent) and several endocytic pathways for intracellular trafficking, a capacity termed “endocytic plasticity” [60], [64]. For instance, diphtheria toxin and the Shiga toxin-related protein toxins (Shiga toxin, ricin, and cholera toxin) enter cells by various clathrin-dependent or -independent mechanisms which can involve raft-type membrane microdomains [59]–[61], [64]–[71]. After entry, the toxins of the Shiga family and *Pseudomonas* ExoA (PE) traffic from the early endosomes to the Golgi apparatus by various routes and bypass the late endosomes/lysosmes stage, which would otherwise lead to degradation [59], [64], [70]–[76].

Several microbes or their toxic proteins exhibit endocytotic trafficking patterns that have similarities to the pattern we described for the *Yersinia* V protein. After gaining entry by endocytosis they fuse with early and late endosomes, and during subsequent inter-organelle migration, induce a chain of events that often results in necrosis. For example, the movement of *Salmonella typhi* in infected cells appears to involve a pattern of vesicular trafficking via endosome and lysosomes [77]. This infection is also associated with the induction of apoptosis, a process requiring the permeabilization of the mitochondrial membrane [58] and which may serve as a possible model for V trafficking. Also, the gastrointestinal pathogen *Helicobacter pylori* exhibits some pathogenic attributes similar to those of the *Yersinia* spp. Observations reported using an *in vitro* model of gastric epithelial cell infection indicate that, like V, *H. pylori* exhibits a pattern of early to late endosomal trafficking. However, the VacA toxin of *H. pylori* causes a disruption of the subsequent events in the endocytic pathway by preventing maturation of late endosomes to lysosomes, a process that likely promotes bacterial survival in the gastric epithelium [60], [63]. The results of studies with a HeLa cell model revealed that VacA also can enter cells, traffic to the mitochondria, and then modulate mitochondrial membrane permeability by a mechanism affecting channel activity. This perturbation results in the release of cytochrome C to the cytosol. These activities are associated with the mitochondrial-mediated apoptotic pathway, and the VacA model represents a novel mechanism for regulation of this pathway by a bacterial toxin [78]. Thus, these systems could potentially provide further insights for our ongoing studies to define V trafficking.

The role played by internalized V antigen in the pathogenesis of plague is unknown. Once candidate V-target interactions are identified, *in vitro* and *in vivo* investigations will be done to analyze the role and importance of the intracellular localization of V in the pathogenesis of infection by *Y. pestis*. The final goal of these studies is to leverage this newly characterized activity of V to develop more specific and effective therapeutics and prophylactics against plague.

## Materials and Methods

### Bacterial strains, reagents, and cell lines

Strains of *Yersinia* used included a pgm-, pPst- cured mutant of the C092 strain of *Y. pestis* (CO92 pPst^−^ pgm^−^) and *Y. pseudotuberculosis* strains YpIII p(IB19), which has an in-frame deletion mutation of the plasmid pLcr-encoded *lcrV* gene, and strain Y. ptb. pTcrV, which is transformed with the recombinant pTrc99A-LcrV expression plasmid [20], [27]; the YpIII p(IB19)-derivative strains were kindly provided by J. Hill. *Y. pseudotuberculosis* strain YpIII p(IB604), harboring an in-frame deletion of the *yopB* gene, was kindly provided by R. Nordfelth and H. Wolf-Watz. Six SMIs of the T3SS, compounds 3, 8, 10–11, 17, and 21 [36], [43] were generously provided by Dr. M. Elofsson at Umea University (Sweden). Monoclonal Abs against V were IgG-purified via high performance liquid chromatography from hybridoma culture supernatants and assessed for binding by ELISA, as described previously [24]. Biotinylated- and FITC-conjugated derivatives of anti-V Ab (clone val3) were obtained from Hytest (Finland). The J774 MΦ-like cell line was maintained in Dulbecco's modified Eagle's minimum essential medium with 10% heated killed fetal bovine serum (FBS). HeLa cells, a human carcinoma epithelioid cell line, were maintained in Roswell Park Memorial Institute medium supplemented with 10–20% heat-inactivated FBS.

In addition to infection, V was translocated into target host cells by utilizing an anthrax toxin PA-LFn system instead of infection (Ribot, unpublished data). The N-terminus region of LF (amino acid residues 1–256 [LFn]) contains the PA binding domain. The fusion protein used in our studies consisted of LFn fused in-frame with the V gene. MФs were washed with 10 mM phosphate buffered saline (PBS) and incubated with 50 µg/ml of LFnV and 3 µg/ml of PA for 4 h at 37°C (5% CO_2_). MФs were fixed for 15 min in 3.7% formaldehyde at 37°C (5% CO_2_), and were examined by IFM, immunoblotting, and flow cytometry.

### Macrophage infection and delivery of V antigen

For IFM and ICM studies, MФs were plated onto Lab-Tek II 4 and 8-well glass chamber slides (Pittsburgh, PA) or Bioptechs Delta T cell culture dishes (Butler, PA). MФs were pretreated for 30 min with 5 ug/ml cytoD, an inhibitor of actin polymerization, and infected with one of the *Y. pestis* or *Y. pseudotuberculosis* strains as described above. In experiments where SMI of T3S were utilized, SMIs were added to *Y. pestis* and *Y. pseudotuberculosis* 30 min before infection to prevent Yops delivery [36], [43]. Samples were taken at 5, 10, 15, 30, and 45 min after infection, and cells were washed three times with PBS. In longer incubations, MΦs were infected for 1 h, washed, then either processed directly for microscopy or incubated with 7.5 µg/ml of gentamicin for an additional 1–5 h for analysis. MΦs were stained, fixed, and permeabilized according to manufacturer recommendations, followed by incubation with diluted mAb to V for 1 h at 37°C (5% CO_2_). Chambers were removed and washed with PBS. All slides were observed under 40×, 60×, and 100×(oil) magnification with a Nikon E800 series fluorescence microscope or with the BioRad 2000MP multiphoton confocal system and a Nikon TE300 inverted microscope.

The effects of mitochondrial inhibition on intracellular V trafficking was monitored by IFM. For the mitochondrial inhibition assays, MΦs were pretreated with 5 µg/ml cytoD, 10 µM Ru360 (Calbiochem, San Diego, CA) [45], or both for 30 min prior to infection with CO92 pPst- pgm- or YpIII p(IB604) strains for 2.5–4 h. For monitoring the trans-Golgi network, some of the MΦs were stained with WGA 1 h before infection. Following infection, MΦs were processed as previously described.

Acquired images were analyzed for co-localization as 8-bit TIFF images in Image J software version 1.40 g (National Institutes of Health, Washington, DC, USA), using the JACoP plugin, which employs a three-dimensional object counter object-based tool in addition to current statistical correlations of intensity [37]. Co-localization was quantified by considering (1) a Pearson's correlation coefficient (r), and (2) a Manders overlap coefficient (r), including a background subtraction. PCC predicts a positive correlation as r approaches 1 and a negative correlation for values close to -1; whereas, MOC indicates 100% co-localization at 1 and no co-localization at 0 [37], [38]. For all markers averages of three experiments were analyzed.

IEM was performed on uninfected MФs or MФs infected with CO92 pPst^−^ pgm^−^ and then fixed in a buffer of 4% paraformaldehyde (Sigma), 0.2% glutaraldehyde (Sigma), and 0.1 M sodium cacodylate (Ricca Chemical Co., Texas) [79]. The samples were then embedded for thin sectioning and stained with gold-labeled Abs to V (6 nm) and organelles (15 nm). Images were taken at a range of 30,000–100,000× using a JEOL JEM 1010 microscope with a Hamamatsu CCD camera.

### Density gradient centrifugation and Immunoblotting

MФs were infected as described above, washed with 4°C PBS, and incubated in a solution of organelle-specific 1× Extraction Buffer (Organelle Isolation Kits®, Sigma Aldrich) and a 0.1% solution of protease inhibitor cocktail (Sigma Aldrich) overnight (ON) on ice at 4°C. Samples were homogenized on ice using a tissue grinder, or processed on a Precellys® 24 Homogenizer (Mo Bio Laboratories, Inc, CA) at two 20 sec cycles of 6500×g using 1.4 mm ceramic beads. Cell lysates were centrifuged at 1000×g for 10 min at 4°C. The post-nuclear supernatants were centrifuged at 3000–3200×g for 30 min at 4°C and the pellets were resuspended in a 0.25 M sucrose homogenization solution containing ioxidanol Optiprep™ (Axis-Shield, Norton, MA). These gradient-forming suspensions were loaded into Quick-Seal polyallomer 13.5 ml tubes (Beckman Coulter, Fullerton, CA), forming a discontinuous gradient. The gradients were centrifuged at 320,000×g in a Beckman VTi65.1 rotor for 3 h at 4°C. One-ml fractions were collected from top to bottom. All fractions were stored at −20°C until further use.

Fractions derived from ultracentrifugation were thawed on ice, combined with lithium dodecyl sulfate (LDS) loading buffer, NuPage reducing agent and antioxidant, and boiled before loading onto replicate 10% NuPage Bis-Tris gels (Invitrogen) with prelabeled Western standards (Invitrogen). The replicate gels were electrophoresed and either stained with SimplyBlue SafeStain (Invitrogen) or the protein bands were transferred to 0.2 µm pore size polyvinylidene difluoride (PVDF) membranes (Invitrogen). Membranes were blocked at 4°C in PBS with 0.2% Tween−20 (Sigma Aldrich) and 7% blotting grade milk (Santa Cruz Biotechnology, Inc.). They were washed with PBS, then incubated 1 h 15 min with a primary Ab listed in Table 1 or with polyclonal Abs to YopM, YopH, YopE, or YopD diluted in 7% blocking solution. Next, the membranes were washed and incubated 1.5 h with secondary Ab conjugated to horseradish peroxidase (HRP). The membranes were washed and incubated in a luminol/peroxide detection solution from the SuperSignal West Pico Chemiluminescence Substrate Kit (Pierce) for 5 min in the dark, exposed to Kodak Biomax ML film, and developed on a Kodak X-OMAT M20 Processor.

### Organelle isolation and Flow cytometry

Before use in flow cytometry, MФs were infected as previously described, washed five times with PBS for 5 min each, and collected into 1× Extraction Buffer with protease inhibitor cocktail (Sigma Aldrich). MФs were observed for the amount of lysis that occurred during the ON incubation by staining with trypan blue, and inadequate lysis (<90%) was remedied by homogenization, as described above; organelles were isolated by using Mitochondria, Endoplasmic Reticulum (ER), Peroxisome, or Lysosome Isolation kits (Sigma Aldrich), according to manufacturer recommendations. Proteins were isolated from the Golgi apparatus using the ER isolation kit. All samples were electrophoresed and transferred as described above, and the blots developed with Ab (Table 1) to each organelle. Isolated organelle-rich samples were pooled and stored at 4°C, −20 °C, or −70°C until used for flow cytometry analysis, according to manufacturer recommendations.

Cell lysates or isolated organelles were fixed, permeabilized, and stained as previously described. Samples were read on a BD Biosciences FACSCalibur (Franklin Lakes, NJ). Regions were drawn in accordance with samples that were stained with the organelle-associate markers and/or with anti-V Ab. Percentages of each region were averaged and entered into GraphPad Prism version 5.01. A one-way ANOVA was performed, followed by paired t-test comparisons of each region (1–3) according to each treatment. For example, organelles stained with anti-V Abs alone were compared with dually-stained organelles. Differences in region 3, in particular, between anti-V Ab stained organelles and those stained with both the organelle-associated markers and anti-V Abs were considered statistically significant at P<0.05.

For cell viability assays, MФs were plated into 24-well cell culture plates (Fisher Scientific) and infected as previously described. The MФs were then washed with PBS, and stained with (1) PI (5 µg/ml) and/or 2.5 mM Syto-13 [80], or (2) DeadRed/Syto-10, according to manufacturer instructions (Invitrogen, Carlsbad, CA) at 37°C for 30 min. Samples were centrifuged at 1000×g, resuspended in PBS, and read on a BD FACSCalibur. PI intercalates into double-stranded nucleic acids and is excluded by viable cells, identifying dead cells by the appearance of red fluorescent staining, as also detected by DEADRed. In contrast, Syto binds nucleic acids of both live cells and appears as green fluorescent staining. Flow cytometry live/dead data from uninfected and infected MФs was entered into GraphPad Prism. A 2-way ANOVA was performed with Bonferroni post hoc tests. Statistically significant differences between strains were considered when P<0.05.

## Supporting Information

Figure S1 Inhibition of V Trafficking as detected by IFM. To determine if V was trafficking from lysosomes to mitochondria and Golgi sequentially or simultaneously, MФs were pretreated with Ru360, an inhibitor of mitochondrial function, cytoD, or both for 30 min before infection with YpIII p(IB604) for 4 h, followed by staining with WGA and anti-V Ab. When cyto D alone was present, co-localization of V with Golgi protein(s) (panel A) was observed, as observed in Fig. 1 (panels E and K). In the presence of Ru360 alone, V staining was too intense to determine localization (panel B). When both cytoD and Ru360 were present (panel C), no co-localization was observed.
(2.10 MB TIF)

Figure S2 The effect of V on cell viability as detected by flow cytometry. MФs were infected with a V- negative strain (YpIII p[IB19]), a T3SS inhibited strain (YpIII p[IB604]), or with intact T3SS and V producing strains (CO92 pPst- pgm- and Y. ptb. pTcrV), with or without SMI of T3S present prior to infection. Percentages of live (stained with Syto) and dead (stained with PI/DEADRed) MФs were plotted. Dead MФs from uninfected samples ranged from ∼9–11% of the total population (data not shown). Infected MФ cell death ranged from 42.24 to 72.67%. Differences between samples were significant by ANOVA (P<0.001). Bonferroni post hoc t-tests were performed comparing all strains to the V-negative strain. Significant differences were observed when MФs infected with the V-negative YpIII p(IB19) strain were compared to those infected with CO92 pPst- pgm- (***, P<0.001) or with Y. ptb. pTcrV (P<0.001); whereas, no significant differences were found when MФs infected with the V-negative strain were compared to those infected with YpIII p(IB604) or with SMI-pretreated CO92 pPst- pgm- infected MФs. However, when Y. ptb. pTcrV was pretreated with SMIs, there was still a significant difference (*, P<0.05) when compared to the V-negative strain.
(0.81 MB TIF)

## References

[1] Butler T (1983). Plague and other *Yersinia* infections..

[2] Perry RD, Fetherston JD (1997). *Yersinia pestis*-etiological agent of plague.. Clin Microbiol Rev.

[3] Perry RD, Straley SC, Fetherston JD, Rose DJ, Gregor J (1998). DNA sequencing and analysis of the low-Ca^2+^-response plasmid pCD1 of *Yersinia pestis* KIM5.. Infect Immun.

[4] Burrows TW, Bacon GA (1958). The effects of loss of different virulence determinants on the virulence and immunogenicity of strains of *Pasteurella pestis*.. Br J Exp Pathol.

[5] Burrows TW (1963). Virulence of *Pasteurella pestis* and immunity to plague.. Ergeb Mikrobiol Immunitatsforsch Exp Ther.

[6] Friedlander AM, Welkos SL, Worsham PL, Andrews GP, Heath DG (1995). Relationship between virulence and immunity as revealed in recent studies of the F1 capsule of *Yersinia pestis*.. Clin Infect Dis.

[7] Galyov EE, Smirnov OY, Karlishev AV, Volkovoy KI, Denesyuk AL (1990). Nucleotide sequence of the *Yersinia pestis* gene encoding F1 antigen and the primary structure of the protein. Putative T and B cell epitopes.. FEBS Lett.

[8] Galyov EE, Karlyshev AV, Chernovskaya TV, Dolgikh DA, Smirnov OY (1991). Expression of the envelope antigen F1 of *Yersinia pestis* is mediated by the product of caf1M gene having homology with the chaperone protein PapD of *Escherichia coli*.. FEBS Lett.

[9] Karlyshev AV, Galyov EE, Abramov VM, Zay'yalov VP (1992). Caf1R gene and its role in the regulation of capsule formation of *Y. pestis*.. FEBS Lett.

[10] Lindler LE, Plano GV, Burland V, Mayhew GF, Blattner FR (1998). Complete DNA sequence and detailed analysis of the *Yersinia pestis* KIM5 plasmid encoding murine toxin and capsular antigen.. Infect Immun.

[11] Parkhill J, Wren BW, Thomson NR, Titball RW, Holden MTG (2001). Genome sequence of *Yersinia pestis*, the causative agent of plague.. Nature.

[12] Welkos SL, Andrews GP, Lindler LE, Snellings NJ, Strachan SD (2004). Mu dI1(Ap lac) mutagenesis of *Yersinia pestis* plasmid pFra and identification of temperature-regulated loci associated with virulence.. Plasmid.

[13] Ferber DM, Brubaker RR (1981). Plasmids in *Yersinia pestis*.. Infect Immun.

[14] Sodeinde O, Subrahmanyam YVBK, Stark K, Quan T, Bao Y (1992). A surface protease and the invasive character of plague.. Science.

[15] Welkos SL, Friedlander AM, Davis KJ (1997). Studies on the role of plasminogen activator in systemic infection by virulent *Yersinia pestis* strain CO92.. Microb Pathog.

[16] Welkos SL, Pitt ML, Martinez M, Friedlander A, Vogel P (2002). Determination of the virulence of the pigmentation-deficient and pigmentation-plasminogen activator-deficient strains of *Yersinia pestis* in non-human primate and mouse models of pneumonic plague.. Vaccine.

[17] Lathom W, Price P, Miller V, Goldman W (2007). A plasminogen-activating protease specifically controls the development of primary pneuominc plague.. Science.

[18] Cornelis GR, Boland A, Boyd AP, Geuijen C, Iriarte M (1998). The virulence plasmid of *Yersinia*, an antihost genome.. Microbiol Mol Biol Rev.

[19] Nilles ML, Fields KA, Straley SC (1998). The V antigen of *Yesinia pestis* regulates Yop vectorial targeting as well as Yop secretion through effects on YopB and LcrG.. J Bacteriol.

[20] Pettersson J, Holmstrom A, Hill J, Leary S, Frithz-Lindsten E (1999). The V-antigen of *Yersinia* is surface exposed before target cell contact and involved in virulence protein translocation.. Mol Microbiol.

[21] Une T, Nakajima R, Brubaker RR (1987). Roles of V antigen in promoting virulence in *Yersiniae*.. Contrib Microbiol Immunol.

[22] Viboud GI, Bliska JB (2005). *Yersinia* Outer Proteins: Role in modulation of host cell signaling responses and pathogenesis.. Annu Rev Microbiol.

[23] Kerschen EJ, Cohen DA, Kaplan AM, Straley SC (2004). The plague virulence protein YopM targets the innate immune response by causing a global depletion of NK cells.. Infect Immun.

[24] Anderson GW, Leary SE, Williamson ED, Titball RW, Welkos SL (1996). Recombinant V antigen protects mice against pneumonic and bubonic plague caused by F1-capsule-positive and -negative strains of *Yersinia pestis*.. Infect Immun.

[25] Nakajima R, Brubaker RR (1993). Association between virulence of *Yersinia pestis* and suppression of gamma interferon and tumor necrosis factor alpha.. Infect Immun.

[26] Nedialkov YA, Motin VL, Brubaker RR (1997). Resistance to lipopolysaccharide mediated by the *Yersinia pestis* V antigen-polyhistidine fusion peptide: amplification of interleukin-10.. Infect Immun.

[27] Powell BS, Andrews GP, Enama JT, Jendrek S, Bolt C (2005). Design and testing for a nontagged F1-V fusion protein as vaccine antigen against bubonic and pneumonic plage.. Biotech Prog.

[28] Weeks S, Hill J, Friedlander A, Welkos S (2002). Anti-V antigen antibody protects macrophages from *Yersinia pestis* -induced cell death and promotes phagocytosis.. Microb Pathog.

[29] Nakajima R, Motin VL, Brubaker RR (1995). Suppression of cytokines in mice by protein A-V antigen fusion peptide and restoration of synthesis by active immunization.. Infect Immun.

[30] Sing A, Roggenkamp A, Geiger AM, Heesemann J (2002). *Yersinia enterocolitica* evasion of the host innate immune response by V antigen-induced IL-10 production of macrophages is abrogated in IL-10-deficient mice.. J Immunol.

[31] Holmstrom A, Olsson J, Cherepanov P, Maier E, Nordfelth R (2001). LcrV is a channel size-determining component of the Yop effector translocon of *Yersinia*.. Mol Microbiol.

[32] Mueller CA, Broz P, Muller SA, Ringler P, Erne-Brand F (2005). The V-antigen of *Yersinia* forms a distinct structure at the tip of the injectisome needles.. Science.

[33] Skrzypek E, Cowan C, Straley SC (1998). Targeting of the *Yersinia pestis* YopM protein into HeLa cells and intracellular trafficking to the nucleus.. Mol Microbiol.

[34] Fields KA, Straley SC (1999). LcrV of *Yersinia pestis* enters infected eukaryotic cells by a virulence plasmid-independent mechanism.. Infect Immun.

[35] Ryning FW, Remington JS (1978). Effect of cytochalasin D on *Toxoplasma gondii* cell entry.. Infect Immun.

[36] Hakansson S, Schesser K, Persson C, Galyov EE, Rosqvist R (1996). The YopB protein of *Yersinia pseudotuberculosis* is essential for the translocation of Yop effector proteins across the target cell plasma membrane and displays a contact-dependent membrane disrupting activity.. EMBO.

[37] Bolte S, Cordelieres FP (2006). A guided tour into subcellular colocalization analysis in light microscopy.. J Microscopy.

[38] Zinchuk V, Zinchuk O, Okada T (2007). Quantitative colocalization analysis of multicolor confocal immunofluorescence microscopy images: pushing pixels to explore biological phenomena.. Acta Histochem Cytochem.

[39] Marketon MM, DePaolo RW, DeBord KL, Jabri B, Schneewind O (2005). Plague bacteria target immune cells during infection.. Science.

[40] Hu H, Sa Q, Koehler TM, Aronson AI, Zhou D (2006). Inactivation of *Bacillus anthracis* spores in murine primary macrophages.. Cell Microbiol.

[41] Cornelis GR (2002). The *Yersinia* Ysc-Yop virulence apparatus.. Int J Med Microbiol.

[42] Marenne MN, Journet L, Mota LJ, Cornelis GR (2003). Genetic analysis of the formation of the Ysc-Yop translocation pore in macrophages by *Yersinia enterocolitica*: role of LcrV, YscF and YopN.. Microb Pathog.

[43] Nordfelth R, Kauppi AM, Norberg HA, Wolf-Watz H, Elofsson M (2005). Small-molecule inhibitors specifically targeting type III secretion.. Infect Immun.

[44] Matlib MA, Zhou Z, Knight S, Ahmed S, Choi KM (1998). Oxygen-bridge dinuclear ruthenium amine complex specifically inhibits Ca^2+^ uptake into mitochondria *in vitro* and *in situ* in single cardiac myocytes.. J Biol Chem.

[45] Sanchez JA, Garcia MC, Sharma VK, Young KC, Matlib MA (2001). Mitochondria regulate inactivation of L-type Ca^2+^ channels in rat heart.. J Physiol.

[46] Griffiths G, Hoflack B, Simons K, Mellman I, Kornfeld S (1988). The mannose 6-phosphate receptor and the biogenesis of lysosomes.. Cell.

[47] Lin SX, Mallet WG, Huang AY, Maxfield FR (2004). Endocytosed cation-independent mannose 6-phosphate receptor traffics via the endocytic recycling compartment en route to the trans-Golgi network and a subpopulation of late endosomes.. MBC.

[48] Alveraz-Dominguez C, Roberts R, Stahl PD (1997). Internalized *Listeria monocytogenes* modulates intracellular trafficking and delays maturation of the phagosome.. J Cell Science.

[49] MacAry PA, Lindsay M, Scott MA, Craig JIO, Luzio JP (2001). Mobilization of MHC class I molecules from late endosomes to the cell surface following activation of CD34-derived human Langerhans cells.. PNAS.

[50] Tuma PL, Nyasae LK, Backer JM, Hubbard AL (2001). Vsp34p differentially regulates endocytosis from the apical and basolateral domain in polarized hepatic cells.. J Cell Biol.

[51] Cataldo AM, Mathews PM, Boyer Boiteau A, Hassinger LC, Peterhoff CM (2008). Down syndrome fibroblast model of Alzheimer-related endosome pathology. Accelerated endocytosis promotes late endocytic defects.. Amer J Pathol.

[52] Feng Y, Press B, Wandinger-Ness A (1995). Rab7: An important regulator of late endocytotic membrane traffic.. J Cell Biol.

[53] Sobo K, LeBlanc I, Luyet PP, Fivaz M, Ferguson C (2007). Late endosomal cholesterol accumulation leads to impaired intra-endosomal trafficking.. PLoS One.

[54] Collier RJ, Young JA (2003). Anthrax toxin.. Annu Rev Cell Dev Biol.

[55] Young JA, Collier RJ (2007). Anthrax toxin: receptor binding, internalization, pore formation, and translocation.. Annu Rev Biochem.

[56] Blanke SR, Milne JC, Benson EL, Collier RJ (1996). Fused polycationic peptide mediates delivery of diphtheria toxin A chain to the cytosol in the presence of anthrax protective antigen.. Proc Natl Acad Sci.

[57] Falnes PO, Sandvig K (2000). Penetration of protein toxins into cells.. Curr Opin Cell Biol.

[58] Jesenberger V, Procyk KJ, Yuan J, Reipert S, Baccarini M (2000). *Salmonella*-induced caspase-2 activation in macrophages: a novel mechanism in pathogen-mediated apoptosis.. J Exp Med.

[59] Johannes L, Decaudin D (2005). Protein toxins: intracellular trafficking for targeted therapy.. Gene Ther.

[60] Johannes L, Goud B (2000). Facing inward from compartment shores: how many pathways were we looking for?. Traffic.

[61] Lord JM, Roberts LM (1998). Retrograde transport: going against the flow.. Curr Biol.

[62] Terebiznik MR, Vazquez CL, Torbicki K, Banks D, Wang T (2006). *Helicobacter pylori* VacA toxin promotes bacterial intracellular survival in gastric epithelial cells.. Infect Immun.

[63] Vonderheit A, Helenius A (2005). Rab7 associates with early endosomes to mediate sorting and transport of Semliki forest virus to late endosomes.. PLoS Biol.

[64] Sandvig K, van Deurs B (2000). Entry of ricin and Shiga toxin into cells: molecular mechanisms and medical perspectives.. EMBO J.

[65] Abrami L, Liu S, Cosson P, Leppla SH, van der Good GF (2003). Anthrax toxin triggers endocytosis of its receptor via a lipid raft-mediated clathrin-dependent process.. J Cell Biol.

[66] Johannes L, Lamaze C (2002). Clathrin-dependent or not: is it still the question?. Traffic.

[67] Morris RE, Gerstein AS, Bonventre PF, Saelinger CB (1985). Receptor mediated entry of diphtheria toxin into monkey kidney (Vero) cells: electron microscopic evaluation.. Infect Immun.

[68] Moya M, Dautry-Varsat A, Goud B, Louvard D, Boquet P (1985). Inhibition of coated pit formation in Hep2 cells blocks the cytotoxicity of diphtheria toxin but not that of ricin toxin.. J Cell Biol.

[69] Ricci V, Galmiche A, Doye A, Necchi V, Solcia E (2000). High cell sensitivity to *Helicobacter pylori* VacA toxin depends on a GPI-anchored protein and is not blocked by inhibition of the clathrin-mediated pathway of endocytosis.. Mol Biol Cell.

[70] Sandvig K, Grimmer S, Iversen TG, Rodal K, Torgersen ML (2000). Ricin transport into cells: studies of endocytosis and intracellular transport.. Int J Med Microbiol.

[71] Shin JS, Abraham SN (2001). Cell biology. Caveolae–not just craters in the cellular landscape.. Science.

[72] Hehnly H, Sheff D, Stamnes M (2006). Shiga toxin facilitates its retrograde transport by modifying microtubule dynamics.. Mol Biol Cell.

[73] Johannes L (2002). The epithelial cell cytoskeleton and intracellular trafficking. I. Shiga toxin B-subunit system: retrograde transport, intracellular vectorization, and more.. Am J Physiol Gastrointest Liver Physiol.

[74] Khine AA, Tam P, Nutikka A, Lingwood CA (2004). Brefeldin A and filipin distinguish two globotriaosyl ceramide/verotoxin-1 intracellular trafficking pathways involved in Vero cell cytotoxicity.. Glycobiol.

[75] Mallard F, Antony C, Tenza D, Salamero J, Goud B (1998). Direct pathway from early/recycling endosomes to the Golgi apparatus revealed through the study of shiga toxin B-fragment transport.. J Cell Biol.

[76] Yu M, Haslam DB (2005). Shiga toxin is transported from the endoplasmic reticulum following interaction with the luminal chaperone HEDJ/ERdj3.. Infect Immun.

[77] Uchiva K, Barbieri MA, Funato K, Shah AH, Stahl PD (1999). A *Salmonella* virulence protein that inhibits cellular trafficking.. EMBO J.

[78] Willhite DC, Blanke SR (2004). *Helicobacter pylori* vacuolating cytotoxin enters cells, localizes to the mitochondria, and induces mitochondrial membrane permeability changes correlated to toxin channel activity.. Cell Microbiol.

[79] Takizawa T, Anderson CL, Robinson JM (2003). A new method to enhance contrast of ultrathin cyrosections for immunoelectron microscopy.. J Histochem Cytochem.

[80] Bashaw J, Norris S, Weeks S, Trevino S, Adamovicz JJ (2007). Development of *in vitro* correlate assays of immunity to infection with *Yersinia pestis*.. Clin Vaccine Immunol.

