# Supplementary figures and images for: In Vitro Intracellular Trafficking of Virulence Antigen during Infection by Yersinia pestis
Source: PLoS One. 2009 Jul 17;4(7):e6281. doi: 10.1371/journal.pone.0006281 (PMC2707630; doi:10.1371/journal.pone.0006281)

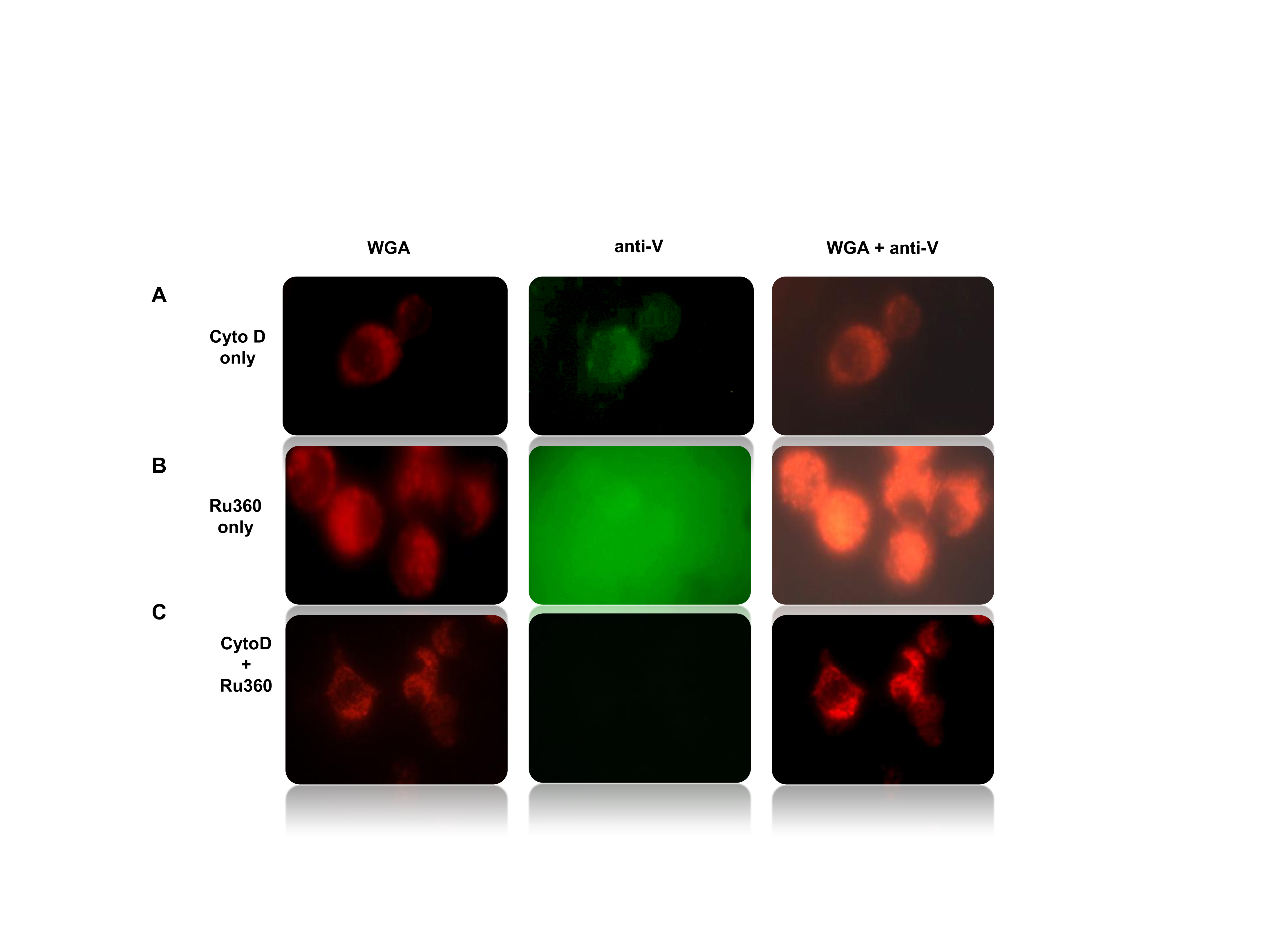

Supplement: Figure S1 — Inhibition of V Trafficking as detected by IFM. To determine if V was trafficking from lysosomes to mitochondria and Golgi sequentially or simultaneously, MФs were pretreated with Ru360, an inhibitor of mitochondrial function, cytoD, or both for 30 min before infection with YpIII p(IB604) for 4 h, followed by staining with WGA and anti-V Ab. When cyto D alone was present, co-localization of V with Golgi protein(s) (panel A) was observed, as observed in Fig. 1 (panels E and K). In the presence of Ru360 alone, V staining was too intense to determine localization (panel B). When both cytoD and Ru360 were present (panel C), no co-localization was observed. (2.10 MB TIF) [file pone.0006281.s001.tif]

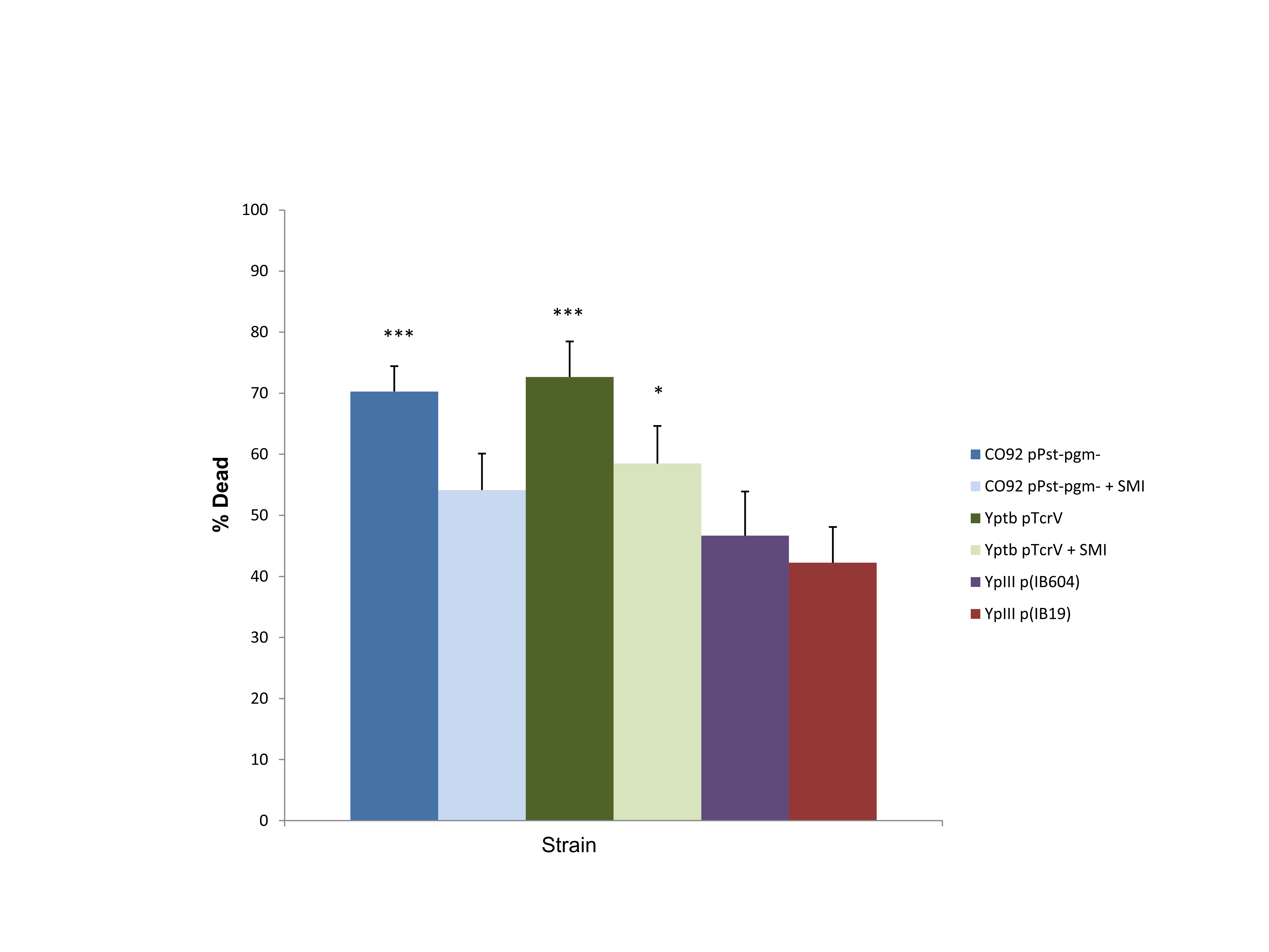

Supplement: Figure S2 — The effect of V on cell viability as detected by flow cytometry. MФs were infected with a V- negative strain (YpIII p[IB19]), a T3SS inhibited strain (YpIII p[IB604]), or with intact T3SS and V producing strains (CO92 pPst- pgm- and Y. ptb. pTcrV), with or without SMI of T3S present prior to infection. Percentages of live (stained with Syto) and dead (stained with PI/DEADRed) MФs were plotted. Dead MФs from uninfected samples ranged from ∼9–11% of the total population (data not shown). Infected MФ cell death ranged from 42.24 to 72.67%. Differences between samples were significant by ANOVA (P<0.001). Bonferroni post hoc t-tests were performed comparing all strains to the V-negative strain. Significant differences were observed when MФs infected with the V-negative YpIII p(IB19) strain were compared to those infected with CO92 pPst- pgm- (***, P<0.001) or with Y. ptb. pTcrV (P<0.001); whereas, no significant differences were found when MФs infected with the V-negative strain were compared to those infected with YpIII p(IB604) or with SMI-pretreated CO92 pPst- pgm- infected MФs. However, when Y. ptb. pTcrV was pretreated with SMIs, there was still a significant difference (*, P<0.05) when compared to the V-negative strain. (0.81 MB TIF) [file pone.0006281.s002.tif]
